# Meeting at Marlborough House

**Published:** 1897-06-05

**Authors:** 


### MEETING AT MARLBOROUGH HOUSE.

A meeting of the General Council of the Prince of Wales' Hospital Fund for London was held at Marlborough House on Friday, the 28th ult., H.R.H. the Prince of Wales in the chair. There were present: The Duke of Norfolk, the Earl of Strafford, the Bishop of London, Lord Rowton, Lord Rothschild (treasurer), Lord Iveagh, Lord Lister, Sir Horace Farquhar, Bart., Sir Arthur Arnold, the Lord Mayor, Rev. J. Guinness Rogers, Rev. T. Bowman Stephenson, Messrs. T. Burt, M.P., Sydney Buxton, M.P., John Aird, M.P., Hugh C. Smith, E. A. Hambro, A. G. Sandeman, Julius Wernher, Dr. Wilks, Sir William MacCormac, and the hon. secretaries (the Right Hon. C. Stuart Wortley, M.P., Sir Savile Crossley, Bart., Mr. Ian Malcolm, M.P., and Mr. J. C. Craggs). Letters of regret at their inability to be present were received from the Duke of Westminster, Cardinal Vaughan, and the Chief Rabbi.

Lord Rothschild reported that the total funds collected to date were in round figures: Annual subscriptions, £19,000; sums given by donors to be invested, £20,000; donations and undefined contributions, £85,000. In addition to these sums the executive hoped to receive £50,000 in respect of the Hospital Stamp, besides contributions from other sources. Of this amount £40,000 had been invested in the names of Mr. Hugh C. Smith, Governor of the Bank of England, and himself. The speaker pointed out that it was obvious that in order to carry out the original intention of the Prince of Wales, renewed efforts were required before June 22nd to secure considerable additions both to annual subscriptions and donations.

H.R.H. the Prince of Wales said: "On the whole we may congratulate ourselves on the result of the subscriptions and donations which we have received up to the present time. Many of us might perhaps have hoped for even more, but I think we must have patience, for many people, I believe, would subscribe if it was pointed out to them how very anxious we now are that not only should this fund be permanently started for the purpose of benefiting the London hospitals, but that this scheme should be one especially commemorating the sixtieth year of the reign of the Queen. (Hear, hear.) Many who have been asked to subscribe I believe have answered, 'Oh, we give annual subscriptions to hospitals—we cannot be called on to give more.' I would only point out to them that if we could induce them to give especially this year to commemorate the Queen's sixtieth anniversary, then I think a great deal more money would flow in, and others would follow their example. (Hear, hear.) I cannot help hoping that some of the great retail trading establishments of the London district will realise more fully than they have done the duty of supporting the London hospitals in consideration of the services made available by the hospitals for the great number of salesmen, saleswomen, clerks, and others whom they employ. I think it will be a source of gratification to you all to know that on informing the Queen that a meeting of the general committee would take place to-day, she empowered me to say that though up till now she has naturally, from a feeling of modesty on her own part, not desired that any suggestion from her should take the place of any given purpose to commemorate her Diamond Jubilee, yet she rejoices to hear what a large sum has already been subscribed to the London Hospital Fund, and wishes the Fund every success." (Hear, hear.) His Royal Highness then read the following letter from Mr. Martin J. Sutton, 107, Lancaster Gate, W., which had been sent to the honorary secretaries:—

"It is with regret I observe that the total sum subscribed to this fund does not form any adequate response to the gracious and earnest appeal made on behalf of the hospitals by His Royal Highness. A multitude of other schemes for commemorating Her Majesty's Diamond Jubilee have since been launched, and the gifts of the Queen's subjects have been diverted to other objects, which, however excellent, are not to be compared in general usefulness and appropriateness to that so happily inaugurated by the Prince of Wales. There remains less than a month in which to carry the Hospital Fund to the success every one desires it should attain. Would it not be possible to make a fresh and very urgent appeal to ensure at least a quarter of a million of money before Jubilee Day? I will gladly give a thousand pounds to the fund on June 22nd, if nine other persons will do the same on or before that date."

The Prince of Wales also read a letter from the Duke of Devonshire, as chairman of the trustees of the Peabody Fund, announcing that the trustees had resolved to give a donation of £10,000 to the fund. His Royal Highness, after mentioning how indebted they were to Mr. Burdett for having started the Hospital stamp, which he had every reason to think would be a great success, continued as follows: "There is only one more subject of importance which I have to bring before you to-day. It must be understood that what I am now going to do is merely to touch on it, for I do not think the moment has arrived to discuss it or to go into it in any detail. Naturally everybody will ask, when we have got a sufficient sum, 'What are you going to do with it? How are you going to spend it?' That is a very grave question, and it will be one for our further careful consideration. Possibly it will be necessary to appoint a small committee to consider the matter and to report to us—the General Committee—what had best be done. It will never do for us to give money right and left to hospitals which we hear are in need. It will be necessary for us to investigate the state in which these hospitals are, to find out those which are in most pressing need of assistance, and also to form some opinion as to the state of efficiency in which they at present exist, and how the management is carried on. I merely mention this now because it must be a subject which will become one of paramount importance in a very short time. I think, however, that it is too soon yet to form any opinion on the subject, which is one which will require most mature consideration. (Hear, hear.) One more matter—I hope that no definite time will be set for the closing of the

Fund. My own opinion is that it should be left open as long as possible, for I believe more and more people will subscribe as the day of the Jubilee approaches, and perhaps even after that." (Hear, hear.)

The Right Hon. C. B. Stuart Wortley, M.P. (one of the hon. secretaries), presented the report of the Executive Committee, which stated that about 180,000 appeals had been sent out by the fund to bankers, limited companies, large firms, residents in the suburbs, &c. The banks had very fully responded to the appeal, and there had been a good response from limited companies. A number of Royal warrant holders had individually subscribed, and the question of a general collection among the holders was being considered by the Association of Her Majesty's Warrant Holders.

The Earl of Strafford formally moved the adoption of the report.

Mr. Sydney Buxton, M.P., in seconding, said he thought they might be well satisfied with the result which had, so far, come from the Prince of Wales's appeal. Some persons outside seemed to think that because the full £100,000 in annual subscriptions had not yet been obtained, that, therefore, the Fund was not a perfect success. He would, however, like to point out that £20,000 in annual subscriptions, and donations amounting to about £100,000, had been promised already, and in regard to the latter sum, though the amounts given were called donations in this particular year, yet still the Fund might look upon a large proportion of them in the light of annual subscriptions, as many of the donors would be induced to continue their donations annually.

The report was adopted.

The Bishop of London congratulated the committee on the cordial relations which the Fund had established with the other societies which were seeking the same object. The greatest securities for the permanence of the Fund were the position it had taken up between the Hospital Sunday and Hospital Saturday Funds, and the fact that it had succeeded in reaching a class of persons who had never before been brought to the support of the hospitals. Referring to the donations received, he said that though a certain number no doubt represented the feelings created by the Jubilee, yet there was a much larger number which represented the gifts of people who had taken up the matter this year, and who, there was every reason to hope, would in future years be equally as likely to continue to give.

The meeting then terminated.

